# Interaction between Fibroblasts and Immune Cells Following DNA Damage Induced by Ionizing Radiation

**DOI:** 10.3390/ijms21228635

**Published:** 2020-11-16

**Authors:** Kalaiyarasi Ragunathan, Nikki Lyn Esnardo Upfold, Valentyn Oksenych

**Affiliations:** 1 Department of Clinical and Molecular Medicine, Norwegian University of Science and Technology (NTNU), 7028 Trondheim, Norway; kalaiyar@stud.ntnu.no (K.R.); nikkiupfold@gmail.com (N.L.E.U.); 2 Department of Clinical Medicine, Faculty of Health Sciences, UiT-The Arctic University of Norway, 9037 Tromsø, Norway; 3 Department of Biosciences and Nutrition (BioNuT), Karolinska Institutet, 14183 Huddinge, Sweden; 4 KG Jebsen Centre for B Cell Malignancies, Institute of Clinical Medicine, University of Oslo, N-0316 Oslo, Norway; 5 Institute of Clinical Medicine, University of Oslo, 0318 Oslo, Norway

**Keywords:** DNA repair, lymphocyte, immune system, DNA breaks, cancer-associated fibroblasts

## Abstract

Cancer-associated fibroblasts (CAF) form the basis of tumor microenvironment and possess immunomodulatory functions by interacting with other cells surrounding tumor, including T lymphocytes, macrophages, dendritic cells and natural killer cells. Ionizing radiation is a broadly-used method in radiotherapy to target tumors. In mammalian cells, ionizing radiation induces various types of DNA damages and DNA damage response. Being unspecific, radiotherapy affects all the cells in tumor microenvironment, including the tumor itself, CAFs and immune cells. CAFs are extremely radio-resistant and do not initiate apoptosis even at high doses of radiation. However, following radiation, CAFs become senescent and produce a distinct combination of immunoregulatory molecules. Radiosensitivity of immune cells varies depending on the cell type due to inefficient DNA repair in, for example, monocytes and granulocytes. In this minireview, we are summarizing recent findings on the interaction between CAF, ionizing radiation and immune cells in the tumor microenvironment.

## 1. Tumor Microenvironment and Radiotherapy

### 1.1. Cancer-Associated Fibroblasts, Tumor Microenvironment and Radiotherapy

Tumor microenvironment (TME) is a combination of tumor cells, immune cells and cancer-associated fibroblasts (CAFs) that interact between each other and with extracellular elements [1]. Radiotherapy (RT) is a powerful, although unspecific, instrument that targets both cancer cells and other elements of the TME, modulating immune response and physiology of CAFs [2]. Non-small cell lung cancer (NSCLC) [3] accounts for about 85% of all lung cancers, being one of the deadliest malignancy types globally [4,5]. Radiotherapy is associated with increased radio-resistance of tumors, including NSCLC, likely due to the pro-tumorigenic activity of CAFs [6]. Pro-tumorigenic nature of irradiated CAFs is explained either by direct stimulation of tumor cell viability or by inhibiting immune cells, such as macrophages, dendritic cells, T cells and natural killers [7,8,9,10,11]. Moreover, one can propose distinct mechanisms of tumor recovery following the therapy and role of CAFs in this scenario. First, the resurgence of tumor due to the malignant cells escaped from the radiotherapy. Second, if all original tumor cells were killed due to the efficient radiotherapy, CAFs and TME could induce de novo tumors. Third, radiotherapy itself damages cells surrounding tumor and some of these cells contribute to de novo tumor growth. In any of these scenarios, the role of CAFs can be significant given their immunosuppressive and tumor-supportive functions [8,10], and needs to be further examined.

**Figure 1:**
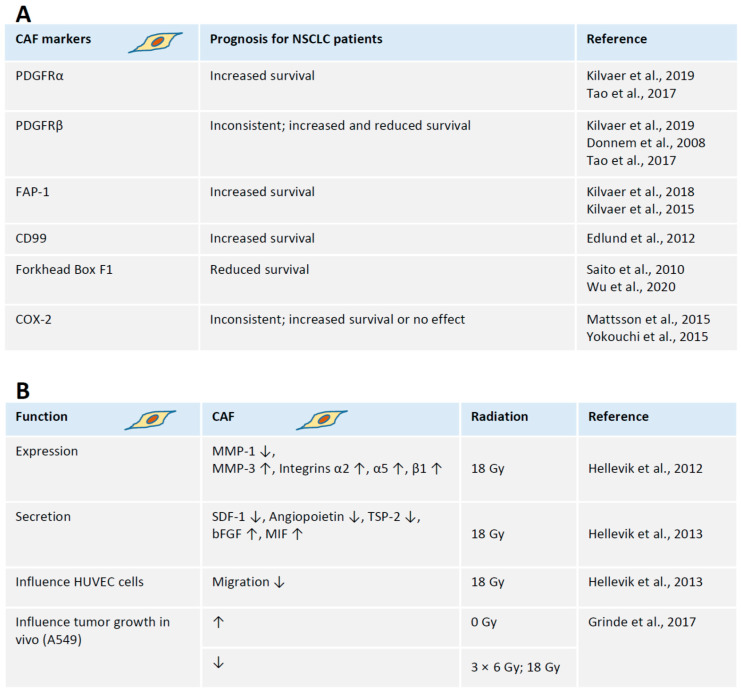
CAFs as a component of tumor stroma. (**A**) Radiation influences CAF physiology and function. (**B**) The prognosis for NSCLC patients based on the CAF biomarkers. References to the Figure 1. Kilvaer et al. [16]; Tao et al., 2017 [19]; Donnem et al., 2008 [20]; Kilvaer et al., 2018 [15]; Kilvaer et al., 2015 [13]; Edlund et al., 2012 [21]; Saito et al., 2010 [22]; Wu et al., 2020 [23]; Mattsson et al., 2015 [24]; Yokouchi et al., 2015 [25]; Hellevik et al., 2012 [12]; Hellevik et al., 2013 [9]; Grinde et al., 2017 [26].

Fibroblasts form a significant part of tumor stroma, and can be defined as cancer-associated fibroblasts (CAF), tumor-associated fibroblasts (TAF), and cancer-associated mesenchymal stem cells (MSC); moreover, fibrosis-associated fibroblasts (FAF) might differ from CAF on a molecular level, suggesting that further research is necessary to characterize specific types and subtypes of fibroblasts in cancer [1]. CAFs, as other fibroblasts, possess spindle-shaped morphology (Figure 1), although gained increased proliferation rates [1]. CAFs are extensively described in literature, including [1,7,8,9,10,12,13,14,15,16,17]. In particular, CAFs can be defined as a heterogenous population of connective tissue cells that contribute to cancer progression by secreting specific molecules, including growth factors, proteases, chemokines and cytokines. These CAF-secreted factors influence adjacent tumor cells, usually inducing tumor growth, as well as attract immune and inflammatory cells [1,10,18]. Due to the different origin and location, multiple cellular markers may assist identifying CAFs, including vimentin, fibroblast-specific protein 1 (FSP1), desmin, discoidin domain-containing receptor 2 (DDR2), αSMA, PDGF receptor-α (PDGFRα), PDGFRβ, FAP, caveolin 1 (CAV1); and secrete vascular endothelial growth factor (VEGF), as well as immunomodulatory molecules, including IL-10, TGFβ, TNF, IFNγ and IL-6 [1].

For radiotherapy in clinics, there are different radiation regimens with total doses ranging from 2 Gy to 74 Gy, which may include single radiation, fractionated, or hypofractionated schedules [27]. In addition to the immunomodulating features of irradiated CAFs, radiotherapy itself enhances the viability of both cancer and associated cells in non-homologous end joining (NHEJ) and DNA damage response (DDR)-dependent manner [28]. There are cons and pros in selected radiation schedules. For example, high doses of radiation (over 10 Gy per time), although result in tumor cell death, anti-tumor signaling and response, lead to severe tissue damage and potential recruitment of immunosuppressive immune cells. Low doses delivered over multiple radiations over weeks (2 Gy and less per time), are less harmful to the tumor itself and result in the recruitment of immune cells, which can be damaged as well over the consequent radiations, reducing benefits of the therapy. Intermediate radiation doses (between 2 Gy and 10 Gy) delivered in several cycles might combine positive effects of high and low dose therapies, and show reduced negative effects [8,10]. Further understanding of mechanisms underlying radiotherapy, particularly the effect on TME, will allow delivering more efficient combinations of radiotherapy with chemo- or immunotherapy [8,10].

### 1.2. Radiotherapy and DNA Damage Response

Ionizing radiation used during the radiotherapy induces DNA breaks, including both single strand (ssDNA) and double-strand DNA (DSB) breaks, which trigger DNA damage response (DDR) [29,30]. Radiation dose determines whether the cell will induce DDR, whether DNA lesions will be repaired, or the cells will never recover from the cell cycle arrest, will enter senescence state or trigger apoptosis. While radiation doses used in clinic vary from 0.1 Gy to 3 Gy, CAFs tolerate relatively high doses of radiation, 30 Gy, without apoptosis, although doses higher than 10–12 Gy result in senescent CAFs [12]. What makes CAFs radioresistant when compared to many other cell types is an unsolved question. One can speculate that CAFs have more efficient DNA repair, more resistant to induce cell cycle arrest via checkpoint proteins, or less prone to trigger apoptosis due to, for example, compromised p53 pathway or high levels of pro-survival Bcl2 family proteins [31,32,33]. Surprisingly, DNA repair efficiency in immune cells may also vary depending on the cell type. While B and T lymphocyte development requires the generation of DSBs during the V(D)J recombination, and B cells have an additional DNA repair-dependent class switch recombination process [34,35,36], monocytes and granulocytes lack certain DNA repair mechanisms [37,38,39,40]. Furthermore, macrophages and dendritic cells re-express DNA repair factors and are resistant to modest levels of DNA damage [38,39,41].

### 1.3. CAFs and NSCLC Prognosis

In a tumor microenvironment, there is a dynamic interaction between components of stroma surrounding cancer cells, and a malignant component [1]. To identify potential CAF markers that can be used for disease prognosis, several studies examined samples from a cohort including 536 to 553 NSCLC patients from Norway and Sweden [13,14,20,42]. In CAFs, high stromal expression of tyrosine kinase receptor, i.e., platelet-derived growth factor receptor (PDGFR)α, correlated with longer survival of patients (stages I–III). Differently, high expression of PDGFRβ had opposite outputs in Sweden (increased survival) and Norway (poor survival), making it not reliable by itself, but potentially useful when multiple other factors are considered [16].

Furthermore, fibroblast activating protein 1 (FAP-1), a marker of fibroblast activation, and a significant marker enabling to distinguish CAFs, is proposed as a biomarker for NSCLC prognosis [13]. High expression of FAP-1 in CAFs correlates with increased disease-specific survival of NSCLC patients [13]. Although high expression of FAP-1 in CAF did not influence the recruitment of tumor-infiltrating lymphocytes (TIFs), the patient’s survival was increased when FAP-1-expressing CAFs are surrounded by high numbers of cytotoxic T cells [15] (Figure 1).

Additional proteins could be relevant prognostic markers, including CD99 [21], Forkhead Box F1 (FOXF1) [22], Cyclooxygenase-2 (COX-2) [24] (Figure 1). In particular, higher stromal expression of CD99 in CAFs correlates with better survival prognosis in a study including materials from 430 NSCLC patients [21]. Based on the study of 247 NSCLC patients, high expression levels of FOXF1 in CAFs is rather a poor survival prognosis [22]. Finally, COX-2 expression in tumor and stromal cells of a large group combining several cohorts and data from 1337 NSCLC patients was used by Mattsson et al. [24]. While COX-2 expression in tumor cells does not correlate with prognosis overall, one cohort suggested a better prognosis for patients with high activity of COX-2 due to expression in stromal cells. It is likely, however, that data on COX-2 need further validation [24].

## 2. Impact of Radiation on CAFs

Following ablative doses of radiation (18 Gy), human CAFs stay alive in tissue culture, although demonstrate persistent DNA damage response (DDR) over days, and rapid senescence [12]. Irradiated CAFs demonstrate reduced migration and invasive capacities in vitro, suggesting changes in the expression of matrix or cytoskeleton proteins. Indeed, irradiated human CAFs possessed reduced levels of matrix metalloproteinase MMP-1 expression, but increased levels of MMP-3 [12] (Figure 1). Moreover, following the ablative radiation of 18 Gy, human CAFs overexpress integrins forming the basis of collagen receptor (α2β1) and fibronectin receptor (α5β1) [12] (Figure 1).

Ablative doses of 18 Gy ionizing radiation result in changes of molecules secreted by CAFs. In particular, irradiated human CAFs release reduced levels of angiogenic molecules, such as stromal cell-derived factor-1 (SDF-1), angiopoietin and thrombospondin-2 (TSP-2) [9]. Moreover, irradiated CAFs release higher levels of fibroblast growth factor bFGF, and macrophage migratory inhibitory factor, MIF. There is no change in expression of hepatocyte growth factor, interleukins IL-6, IL-8, IL-1β and tumor necrosis factor TNFα [9]. Furthermore, the factors released by irradiated CAFs inhibit the migratory capacity of human umbilical vein endothelial cells (HUVECs), suggesting a beneficial therapeutic effect of ablative doses radiation based on the in vitro study [9] (Figure 1).

In vivo models demonstrate that irradiation of CAF (iCAF) changes protumorigenic features, reducing tumor engraftment and angiogenesis [26]. For example, CAFs facilitate tumor growth when co-transplanted into athymic nude mice together with human cancer cells. However, CAFs pretreated with either single 18 Gy or fractionated 3 × 6 Gy radiation regimens, are unable to stimulate tumor growth [26]. Of note, implanted fibroblasts, both CAFs and iCAFs, are detected only in mice during the first week following transplantation, and no longer detected during the weeks two to four, suggesting that the transplanted CAFs and iCAFs gradually die in situ during the first days of experiment [26] (Figure 1). It is possible that CAFs stimulate initial engraftment and growth of tumor cells and are less important at the later stages of carcinogenesis. It is also likely that those human CAFs are replaced by murine CAFs to continue maintaining tumor microenvironment in the trans-species experiments, and further in vivo experiments in mice using only murine cells, both CAFs and tumors, can be considered to figure out these aspects of tumorigenesis in real-time.

## 3. Impact of Radiation on Immune Cells

### 3.1. Radiation and T Cells

**Figure 2:**
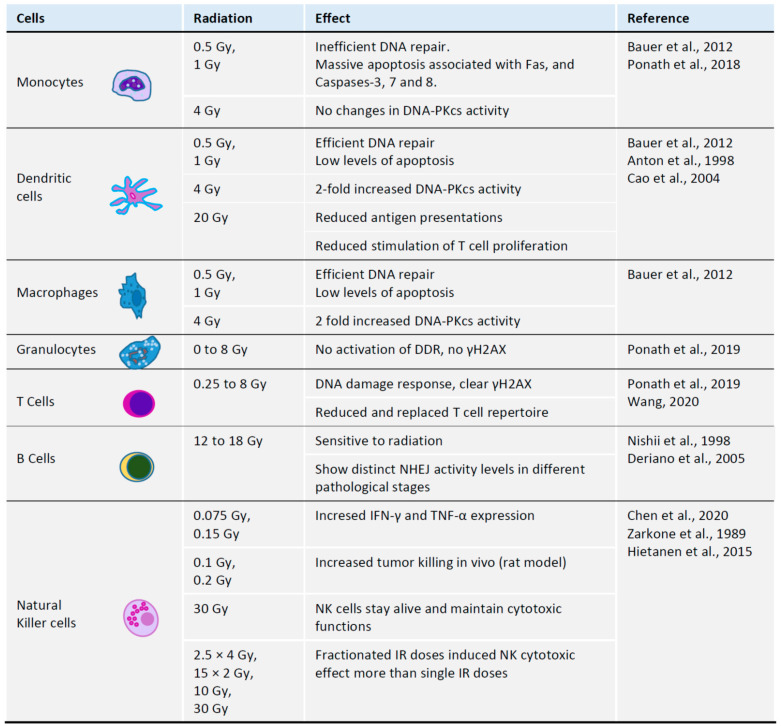
Impact of radiation on monocytes, dendritic cells (DC), macrophages, granulocytes, T cells and natural killer (NK) cells in human model systems, unless specified otherwise. Indicated irradiation doses are in range of clinically-relevant doses, depending on the regimens. References to the Figure 2. Bauer et al., 2012 [40]; Ponath et al., 2018 [41]; Anton et al., 1998 [46]; Cao et al., 2004 [47]; Ponath et al., 2019 [37]; Wang et al., 2020 [43]; Nishii et al., 1998 [48]; Deriano et al., 2005 [49]; Chen et al., 2020 [50]; Zarkone et al., 1989 [51]; Hietanen et al., 2015 [52].

The outcomes of anti-tumor therapies and immune responses are heterogeneous, potentially due to the “holes” in T cell receptor repertoires, in addition to the variation of major histocompatibility complexes and tumor neo-antigens [43]. Moreover, radiotherapy is toxic for T cells and likely for hematopoietic progenitors that could be used to reconstitute T cell populations [44]. Following ionizing radiation, T cell numbers may recover, although their repertoires cannot be restored. Unlike T helper and T cytotoxic cells, regulatory T cells (Treg) are comparatively radioresistant [45]. Changes in T cell receptor repertoire is also expected with age, making the immune system changes even more dynamic and unpredictable when older patients undergo radiotherapy [43,44] (Figure 2).

### 3.2. Radiation and B Cells

B cells and their precursor cells are hypersensitive to the irradiation-induced DNA damage [48]. However, using the C57BL/6 mouse model treated with 12–18 Gy of focal radiation to the tumor site, it was shown that radiation turns B cells to act against tumorigenesis and alters B cell activation, differentiation and clonality [53]. Irradiation induces B cell maturation and activation, as well as increases differentiation of plasma cells specific for tumor antigens [53]. Furthermore, ionizing radiation induces expression of CD20, which is a surface antigen found on the large proportion of the B-lymphomas and used as a target in distinct therapy strategies, such as radio-immunotherapy and antibody-based therapy [54]. Moreover, the cells present in distinct B cell lymphomas and leukemias exhibit various levels of radiosensitivity. Notably, radioresistance has been observed in B lymphoblastoids when compared to normal B cells, although the Burkitt’s lymphoma cells showed hypersensitivity to irradiation [55]. Further, in the B cell chronic lymphocytic leukemia (B-CLL), radioresistant and sensitive cell populations have been reported with distinct levels of NHEJ activities [49].

### 3.3. Radiation and Monocytes

Monocytes are immune cells that differentiate into macrophages and myeloid lineage dendritic cells (DC). Monocytes are hypersensitive to ionizing radiation and oxidative damages resulting in single- and double-strand DNA breaks [39]. Monocytes lack or have low expression of DNA repair proteins, such as X-ray cross complementing factor 1 (XRCC1), DNA ligase III (LIG3), poly-ADP-ribose polymerase 1 (PARP1), and DNA-dependent protein kinase, catalytic subunit (DNA-PKcs), affecting base excision repair (BER) and non-homologous end-joining (NHEJ). Both macrophages and dendritic cells, however, upregulate these factors and show relatively normal DNA repair damage response and DNA repair [39]. Monocytes with damaged DNA activate DNA damage response that includes ATM, ATR, Chk1, Chk2 and p53. Stabilized p53 triggers apoptosis associated with the upregulation of death receptor Fas and activated caspases 3, 7 and 8 [39]. Clinically-relevant doses of 0.5 Gy and 1 Gy ionizing radiation are tolerated by dendritic cells and macrophages that efficiently repair DNA lesions. However, doses of 0.5–1 Gy induce massive apoptosis of monocytes associated with inefficient DNA repair (Figure 2) [39]. Lack of DNA-PKcs in human cells results in more severe defects in NHEJ-mediated DNA repair than in mice [34,35,56,57], which can be explained by lower redundancy, and these data in human cells, although clinically relevant, might not be identical if experiments are performed using mouse models. Moreover, lack of PARP1, LIG3 and XRCC1 potentially abrogates alternative end-joining, further reducing the efficiency of DSB repair in monocytes [39,58]. Higher doses of irradiation (20 Gy) affect functions of dendritic cells, resulting in lower efficiency of antigen presentation [46] and lower capacity to induce proliferation of T lymphocytes [47].

### 3.4. Radiation and Granulocytes

**Figure 3:**
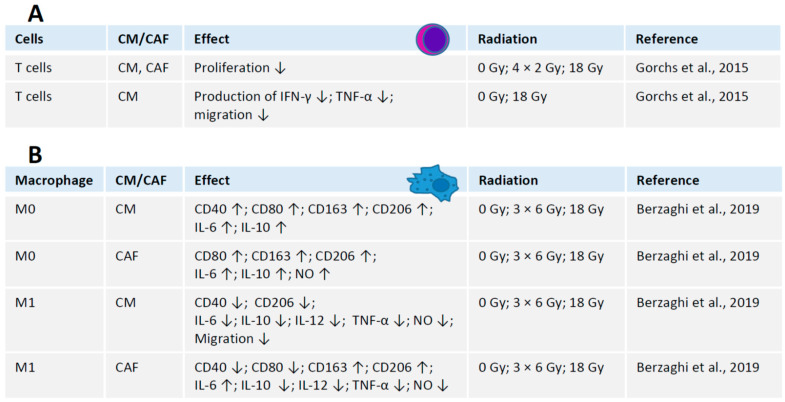
Interaction between CAFs, immune cells and radiation. (**A**) Interaction between irradiated CAFs and T cells. (**B**) Interaction between irradiated CAFs and macrophages. M0, resting macrophages; M1, polarized activated pro-inflammatory macrophages. CAF, co-culture with cancer-associated fibroblasts; CM, conditioned medium from cancer-associated fibroblasts. Summarized from Gorchs et al., 2015 [11] and Berzaghi et al., 2019 [7].

Granulocytes, mainly neutrophils, or polymorphonuclear neutrophilic granulocytes, arise from the same precursor as monocytes and possess similar DNA repair defects to monocytes (Figure 3) [37]. Resembling monocytes, granulocytes also lack key DNA repair factors XRCC1, LIG3, PARP1 and DNA-PKcs. Furthermore, a unique feature of granulocytes is lack of ATM, ATR and inability to phosphorylate histone H2AX (γH2AX). DNA damage-dependent apoptosis is detected in T cells/PBL, but not in granulocytes (Figure 2) [37].

### 3.5. Radiation and Natural Killer Cells

Whole-body irradiation with doses higher than 1 Gy results in acute radiation syndrome, and doses higher than 2 Gy lead to massive death of lymphocytes and hematopoietic progenitors, resulting in hematological crisis [59]. While low doses of IR activate NK cells, higher doses impair NK functions [50]. Roles of NK cells are determined by activating and inhibiting receptors. Low doses of IR, such as 0.075 Gy to 0.15 Gy, trigger increased expression of IFN-γ and TNF-α in vitro, and doses of 0.1 Gy to 0.2 Gy result in NK activation in rat models in vivo (reviewed in [50]). IR induces ATM-dependent DNA damage response in NK, which may facilitate immune response and reduce exhaustion [60]. Higher doses of IR may be tolerated by IL-2 pre-treated NK cells, which maintain their cytotoxic functions [51]. Fractionated doses of 4 × 2.5 Gy, as well as 2 × 15 Gy, resulted in higher NK cytotoxicity than single doses, such as 30 Gy or 10 Gy [52].

## 4. Crosstalk between Radiation, CAFs and Immune Cells

Unlike normal fibroblasts, CAFs suppress the immune response in tumor microenvironment [61]. High levels of CAFs in tumors are associated with poor treatment outcome and prognosis [18,62,63]. Whether radiotherapy affects the interaction between CAFs and tumor cells was recently studied using several model systems, such as T cells [11] and macrophages [7]. It would be also important to investigate the relationship between radiated CAFs and other immune system cells, including but not limited to dendritic cells and natural killers.

### 4.1. Interaction between Irradiated CAFs and T Cells

Both iCAFs and intact CAFs possess immunosuppressive effects, i.e., by reducing proliferation rates of human T cells [11]. Moreover, culture medium from irradiated or intact CAFs has the same immunosuppressive effect, suggesting that it depends on regulatory molecules secreted by CAFs to tumor microenvironment [11] rather than on physical interaction between the cells. Furthermore, CAFs suppress the production of regulatory molecules by T cells, including interferon-gamma (IFN-γ) and tumor necrosis factor alpha (TNF-α) [11]. Both CAFs and iCAFs block migration capacity of T cells (Figure 2) [11].

### 4.2. Interaction between Irradiated CAFs and Macrophages

Macrophages are a part of the tumor microenvironment that interacts with CAFs and tumor cells. While CAFs influence both stimulated (M1) and unstimulated (M0) human macrophages in vitro, irradiation (18 Gy) does not affect these interactions [7]. Medium containing molecules secreted by CAFs, or conditioned medium, stimulates expression of CD40, CD80, CD163, CD206, IL-6 and IL-10 in M0 macrophages. Co-culture with CAFs stimulates M0 macrophages to produce CD80, CD163, CD206, IL-6, IL-10, and nitric oxide (NO) (Figure 3). In contrast, M1 macrophages treated with CAF conditioned medium produce less CD40, CD206, IL-6, IL-10, IL-12, TNF-α, and nitric oxide. Moreover, molecules secreted by CAF abrogate migration of M1 macrophages [7,64] (Figure 3).

Co-culture of irradiated or intact CAFs induces M0 macrophages to produce higher levels of CD80, CD163, CD206, IL-6, IL-10, and nitric oxide (Figure 3). Again, CAFs force M1 macrophages to produce less CD40, CD80, IL-10, IL-12, TNF-α and nitric oxide. However, the expression of CD163 and CD206 is increased in M1 macrophages co-cultured with CAFs (Figure 3). In summary, factors secreted by CAFs inhibit the pro-inflammatory functions of M1 macrophages [7] (Figure 3).

### 4.3. Interaction between CAFs and Other Immune Cells in Radiation Context

Dendritic cells (DC) represent key immune anti-tumor response [2,65]. Tryptophan-2,3-dioxygenase (TDO2), IL-6 and thymic stromal lymphopoietin secreted by CAFs abrogate differentiation and functions of DC [66], resulting in increased expression of IL-10 and TGF-β, reduced expression of CD1a, CD80, CD86, HLA-DR by DCs, infiltration of immunosuppressive regulatory T cells [67], and inability to stimulate differentiation of T cells into T helper type 2, Th2 [68]. It would be of interest to follow on the impact of radiated CAFs on the differentiation of monocytes to dendritic cells, including expression of specific surface markers, as well as focus on the interaction between dendritic cells and T cells in the presence of irradiated CAFs, or conditioned CAF medium.

Natural killer cells are immune system effectors capable to kill, for example, tumor cells and cells infected by viruses [69]. NK communicates with the components of the tumor microenvironment, including CAFs, dendritic cells and macrophages [70]. It would be of interest to investigate how irradiated CAFs influence cytotoxicity of NKs, and both activating and repressing functions of NK cells.

## 5. Hypoxia and CAFs

Oxygen level is one of the factors determining cellular response to irradiation, and hypoxia leads to about three-fold increased levels of radioresistance in the cells [71]. In TME, intratumoral hypoxia is one of the major reasons for the dysfunctional neovasculature. CAFs, which is the predominant cell type present in the tumor stroma, are involved in angiogenesis by secreting various pro- and anti-angiogenic factors [72]. CAFs adapt to local hypoxia by changing metabolism and increasing, for example, glycolysis, catabolic activity, autophagy, as well as enhancing the VEGF signaling [1,17,72,73,74]. For instance, CAFs were reported to deregulate glucose metabolism following epigenetic reprogramming and thus facilitating the progression of breast cancer [17]. In particular, hypoxia and hypoxia-inducible factor 1 alpha (HIP1α) modulate CAFs metabolism in both mice and humans; moreover, hypoxia results in normal fibroblasts to reprogram transcription and to gain CAF-like features [17]. Moreover, in colorectal cancer, hypoxia was shown to change the CAFs metabolism, which resulted in higher levels of TGF-β2 expression and thus chemotherapy resistance of tumor [75]. Hypoxia was shown to trigger breast cancer growth supported by CAFs [76]. Overall, hypoxic conditions and subsequently adjusted metabolic pathways of CAFs can be taken into consideration while developing new therapeutic strategies.

## 6. Conclusions and Future Directions

**Figure 4:**
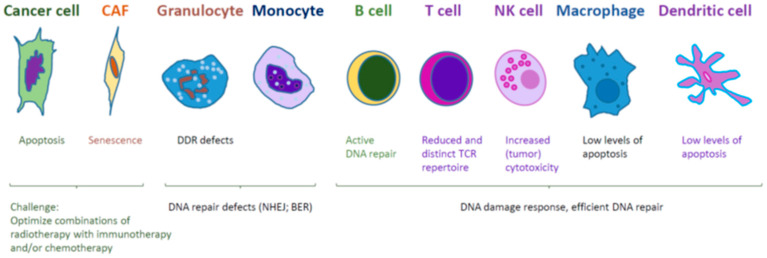
Summary. Effects of radiation on cancer cells, CAFs and immune cells. Combination of immunotherapy and radiotherapy, or a combination of different radiation regimens, is a possible direction to improve treatment efficiency. Depletion of CAFs by combining radiotherapy and chemo- or immunotherapy might reduce pro-tumorigenic and immunosuppression activities. Monocytes and granulocytes lack NHEJ and BER DNA repair factors and hypersensitive to radiation. B cells, T cells, NK cells, macrophages and dendritic cells possess efficient DDR and DNA repair and show distinct responses to radiation.

The studies of the interaction between radiation, CAFs and immune cells are well in progress. To get further in this road, it would be necessary to focus on various immune cells, including progenitor cells of different lineages and even hematopoietic cells (Figure 4). It would be possible to include different radiation schedules, such as low (less than 2 Gy), medium (2 Gy to 10 Gy) and high doses (more than 10 Gy). Further, it would be necessary to consider both traditional in vitro and in vivo research models, including cell lines, mice and rats, as well as more modern 3D cell cultures and organoid systems, in combination with modern imaging techniques.

Furthermore, it is likely that radiotherapy will be combined with chemotherapy and immunotherapy. Whether elimination or depletion of CAFs is beneficial for the overall successful outcome of the therapy is an intriguing and open question. One challenge is unusual radioresistance of CAFs that do not die at doses up to 30 Gy, but instead became senescent and maintain pro-tumorigenic and immunosuppressive capacities [1,7,8,9,10,12,18]. The B cell lymphoma 2 (Bcl-2) family of proteins includes factors that prevent apoptosis, for example, Bcl-xL, Bcl-2 and Mcl-1 [77]. A number of Bcl inhibitors with potential clinical applications have been developed, such as ABT-737 and its derivatives, including navitoclax (ABT-263) and venetoclax (ABT-199); WEHI-539 and its derivatives (A-1331852 and A-1155463); A1210477, S55746, S63845 and S64315. While ABT-199 has been already approved for clinical usage, several other Bcl-2 inhibitors are currently in clinical trials as potential agents for cancer chemotherapy, including ABT-263 and S63845 [77]. Thus, to eliminate CAFs from the radiated tumor microenvironment, Bcl2 inhibitors, including A-1155463 and ABT-199, and existing anticancer drugs can be used, for example, amsacrine, SN38, cisplatin, mitoxantrone, dactinomycin, dinaciclib, UCN-01, bortezomib, and S63845 [77,78,79,80,81,82].

Intratumoral hypoxia is one of the principal reasons for radioresistance. Hypoxia leads to remodeling of the CAFs expression profiles that in return facilitates angiogenesis at TME. Neovasculature and tumor-associated modifications induced by CAFs ultimately contribute to tumor progression, metastasis and diminish therapeutic efficacy of treatment regimens.

Future tumor treatment regimens might combine radio-, chemo- and immunotherapy, and specifically target CAFs in addition to cancer cells.

## Abbreviations

bFGF	Basic fibroblast growth factor
CAF	Cancer-associated fibroblasts
CD	Cluster of differentiation
CM	Conditioned medium
COX-2	Cyclooxygenase-2
DC	Dendritic cells
DDR	DNA damage response
FAP-1	Fibroblast activating protein 1
Gy	Gray, unit of ionizing radiation in the International System of Units
HLA	Human leukocyte antigen
HUVEC	Human umbilical vein endothelial cells
iCAF	Irradiated cancer-associated fibroblasts
IFN	Interferon
IL	Interleukin
IR	Ionizing radiation
M0	Resting macrophage
M1	Activated (polarized) pro-inflammatory macrophage
MIF	Macrophage migration inhibitory factor
MMP	Matrix metalloproteinase
NHEJ	Non-homologous end joining
NK	Natural killer cell
NO	Nitric oxide
NSCLC	Non-small cell lung cancer
PDGFR	Platelet-derived growth factor receptor
SDF-1	Stromal cell-derived factor 1
TGF	Transforming growth factor
TIF	Tumor-infiltrating lymphocytes
TME	Tumor microenvironment
TNF	Tumor necrosis factor
TSP-2	Thrombospondin-2
VEGF	Vascular endothelial growth factor
Y705	Tyrosine 705
